# Expression of an (Engineered) 4,6-α-Glucanotransferase in Potato Results in Changes in Starch Characteristics

**DOI:** 10.1371/journal.pone.0166981

**Published:** 2016-12-02

**Authors:** Xuan Xu, Annemarie Dechesne, Richard G. F. Visser, Luisa M. Trindade

**Affiliations:** 1 Wageningen UR Plant Breeding, Wageningen University and Research, P.O. Box 386, 6700, AJ, Wageningen. The Netherlands; 2 National Centre for Vegetable Improvement (Central China), Key Laboratory of Horticultural Plant Biology, Ministry of Education, Huazhong Agricultural University, Wuhan, 430070, China; National Taiwan University, TAIWAN

## Abstract

Starch structure strongly influences starch physicochemical properties, determining the end uses of starch in various applications. To produce starches with novel structure and exploit the mechanism of starch granule formation, an (engineered) 4, 6-α-glucanotransferase (GTFB) from *Lactobacillus reuteri* 121 was introduced into two potato genetic backgrounds: amylose-containing line Kardal and amylose-free mutant *amf*. The resulting starches showed severe changes in granule morphology regardless of genetic backgrounds. Modified starches from *amf* background exhibited a significant increase in granule size and starch phosphate content relative to the control, while starches from Kardal background displayed a higher digestibility, but did not show changes in granule size and phosphate content. Transcriptome analysis revealed the existence of a mechanism to restore the regular packing of double helices in starch granules, which possibly resulted in the removal of novel glucose chains potentially introduced by the (engineered) GTFB. This amendment mechanics would also explain the difficulties to detect alterations in starch fine structure in the transgenic lines.

## Introduction

Starch, the most important source of calories in human diet, is the major storage carbohydrate in various photosynthetic tissues and storage organs. Starches from different botanic origins vary in granule shape and size, starch composition and structure as well as their properties. Notwithstanding these differences, the composition of native starches is universally composed of amylopectin (70–80%) and amylose (20–30%) with some minor components such as lipids and proteins (reviewed in [1]). Amylose is mostly a linear molecule formed by a-1,4-linked glucose residues and less than 1% α -1,6 branching points, whereas amylopectin is a highly branched molecule with 4–5% α -1,6 linkages. These two components are packed in ordered arrays within the granule, giving rise to alternating semicrystalline and amorphous growth rings [2]. However, mechanisms underlying starch granule formation remain unclear, especially in storage starches.

Over the past decade, *in planta* production of starches with novel properties using genetic modification has attracted particular attention, as it potentially generates environmental and economic benefits and broadens starch end-uses in industrial applications [3–5]. Many studies focused on the alteration in starch structure due to its great effect on starch properties, such as gelatinization properties, swelling power, pasting properties [6–8]. Efforts have been made to introduce changes in amylopectin fine structure by modulating endogenous gene expression in different species [9–13]. For instance, simultaneous downregulation of two starch synthases (SSII and SSIII) in potato resulted in enrichment in shorter chains and a depletion in longer chains of amylopectin, which ultimately affects starch gelatinization temperature and viscosity [14]. Moreover, downregulation of three starch synthases (GBSSI, SSII and SSIII) generated an amylose-free starch with short-chain amylopectin, which showed high freeze-thaw stability [15]. On the other hand, expression of heterologous genes in potato has proven to have great potential to modify starches *in planta* [1]. These genes may have properties that are slightly different from their plant counterparts and thus create different or novel phenotypes. An example of this is the study carried out by Kortstee et al. [16] in which *Escherichia coli* glycogen branching enzyme has been introduced in amylose-free potato mutant, resulting in 25% higher branching degree of amylopectin.

A 4,6-α-glucanotransferase from *Lactobacillus reuteri* 121 (GTFB) is a novel enzyme that can convert starch or starch hydrolysates into isomalto/maltopolysaccharides (IMMPs) [17]. This enzyme can transfer the non-reducing glucose moiety of an α-1,4 glucan chain to the non-reducing end of another α -glucan through α-1,6 linkages, generating a linear chain with α-1,6 linkages [18,19]. This specific activity makes GTFB an interesting target enzyme for producing novel starches *in planta*.

In this study, GTFB either alone or fused to a starch-binding domain (SBD) has been introduced into amylose-containing (Kardal) and amylose-free mutant (*amf*) potato genetic backgrounds. The effects of the (engineered) GTFB on starch characteristics and starch biosynthetic pathway are presented and discussed.

## Materials and Methods

### Construction of plasmids

**Fig 1:**
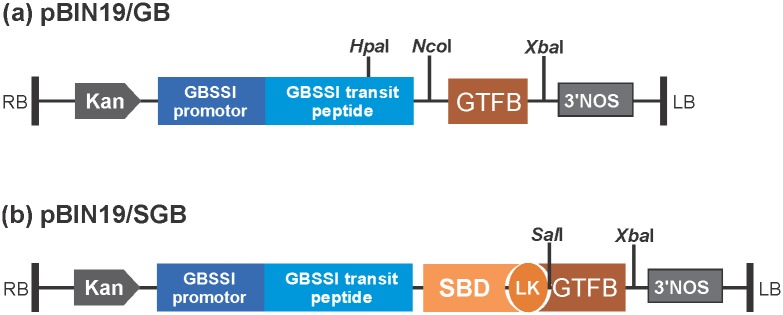
Schematic depiction of two different binary vector constructs (a) pBIN19/GB and (b) pBIN19/SGB. Genes were cloned in frame with GBSSI transit peptide to allow amyloplast targeting and were driven by the potato GBSSI promoter for tuber expression. RB and LB represent right and left borders, respectively. SBD, LK, Kan and 3’NOS stand for starch binding domain of cyclodextrin glycosyltransferase from *Bacillus circulans*, linker, kanamycin resistant gene and NOS terminator, respectively. *Sal*I, *Xba*I, *Hpa*I, *Nco*I and *Xba*I are restriction enzymes.

*GTFB* was amplified from genomic DNA of *Lactobacillus reuteri* 121 with the forward primer F (5′–ATGGAACTCAAAAAACATTTTAAGC–3′) paired with the reverse primer R (5′–TTAGTTGTTAAAGTTTAATGAAATTG–3′). Two constructs were made in this study (Fig 1). One construct, pBIN19/GB, was used for the expression of GTFB; another construct, pBIN19/SGB, was used for the expression of fusion protein, in which GTFB was fused in-frame to the starch-binding domain (SBD) of *Bacillus circulans* cyclodextrin glycosyltransferase at the carboxyl terminus.

The pBIN19/GB plasmid was constructed by using the amplified fragment to substitute the *SBD2* fragment in the pBIN19/SBD2-*XbaI* vector (S1 Fig). The fragment consisted of two parts: a part of transit peptide and full-length *GTFB* gene. To generate this fragment, two starting fragments with overlapping ends were amplified with the forward primer TP-F (5′–ATA**GTTAAC**AAGCTTGATGGGCTCCAATC–3′) paired with the reverse primer TP-R (5′–CTTAAAATGTTTTTTGAGTT**CCATGG**TACAAACAATGGTAGCTGAG–3′), and forward primer GB-F (5′-AGCTACCATTGTTTGTA**CCATGG**AACTCAAAAAACATTTTAAGC–3′) paired with the reverse primer GB-R (5′–CT**TCTAGA**TTAGTTGTTAAAGTTTAATGAAATTGCAG–3′). These PCR products were mixed in a final PCR reaction to generate the full-length fragment with primers TP-F and GB-R. The resulting fragment was digested with *Hpa*l and *Xba*I and ligated sequentially into pBIN19/SBD2-*XbaI* to generate pBIN19/GB (Fig 1a).

To generate the pBIN19/SGB plasmid, a *GTFB*-encoding fragment was obtained by PCR amplification with the primers 5′–GC**G****TCGAC**ATGGAACTCAAAAAA–3′ and 5′–CTAG**TCTAGA**TTAGTTGTTAAAGTTTAATGAAATT–3′, which contained *Sal*I and *Xba*I sites at their 5' ends, respectively. This amplified *GTFB* fragment was digested with *Sal*I and *Xba*I and ligated into pBIN19/SBD2-*XbaI* vector, generating the pBIN19/SGB construct (Fig 1b).

### Transformation and regeneration

Four-week-old shoots from amylose-containing potato (cv. Kardal, tetraploid) and amylose-free mutant (*amf*, diploid) were used for *Agrobacterium*-mediated transformation [20]. In this study, transgenic plants were labelled KDGBxx, KDSGBxx, *amf*GBxx and *amf*SGBxx in which KD and *amf* represent Kardal and *amf* background, GB and SGB stand for GTFB and SBD-GTFB, respectively, xx represents the number of the transgenic line. Transgenic shoots were selected on a rooting medium containing kanamycin at concentrations of 100 mg/L, followed by PCR-based selection using primers specific for the kanamycin resistance gene (*NPTII*) and the target gene (*GTFB*). Ultimately, 32, 27, 28 and 19 independent plantlets from KDGB, KDSGB, *amf*GB and *amf*SGB series, respectively, were generated. Five clones of each line were multiplied by culturing nodal explants according to the procedure described in [20]. These transformants as well as control plants (untransformed plants and transformed plants with empty vector) were further grown in the greenhouse under standard greenhouse conditions (16 h light at 20°C and 8 h dark at 18°C) to generate mature tubers. No differences were detected between untransformed lines and transformed plantlets with empty vector, therefore untransformed lines (UT-KD and UT-*amf*) were further used as controls.

### Starch isolation

To minimize individual variation, tubers from five plants from the same clone were pooled together, and starch isolation was performed according to the procedure described in [21].

### Western dot blot analysis

Fifty mg of starch were heated at 100°C for 5 min with 400 μl of a 2 × SDS sample buffer containing 5% β-mercaptoethanol [22]. After cooling to room temperature, the supernatant was transferred to a 96-well format dot-blot manifold (Schleicher & Schuell, Keene, NH). The dot-blot manifold was connected to a water pump, and a vacuum was applied for 5 min until all samples were impacted on the nitrocellulose membrane (Bio-rad).

The dot blots were blocked overnight at 4°C in Tris-buffered saline (TBS is 20 mM Tris, 500 mM Nacl pH 7.5) with 0.1% Tween-20 (TBST) containing 5% (w/v) dry powdered non-fat milk. Membranes were then incubated for 2 h at room temperature with a 1:1000 dilution of the anti-SBD antibody [23] in 3% non-fat milk in TBST, followed by 5 rinses in TBST. The membrane was then incubated with a 1:5000 dilution of horse radish-peroxidase-conjugated anti-(rabbit IgG) (Cat # A0545, Sigma) in TBST buffer with 3% (w/v) of non-fat milk for 1 h at room temperature. After rinsing 5 times in TBST, SBD-GTFB was detected with a West Femto supersignal (Cat # 34094, Thermo Scientific).

### Analysis of starch characteristics

All analyses conducted in this study have been performed in duplicate unless indicated otherwise.

Particle size distribution and gelatinization properties of starches were analysed with Coulter Multisizer II (Beckman-Coulter, UK) and Differential Scanning Calorimetry (DSC), respectively. Samples were analysed according to the procedure used by Ji et al. [24].

Amylopectin chain length distribution was determined by using high-performance anion-exchange chromatography with pulsed amperometric detection (HPAEC-PAC). Degree of polymerisation (DP) 6–35 were separated according to the method described in Huang et al. [21]. To detect linear α-1, 6 chains in starch structure, 5 mg of starch was suspended in 250 μl of DMSO and gelatinized for 15 min at 99°C. After cooling down, 745 μl of NaAc buffer (100 mM, pH 5.6) was added, followed by incubation with a sufficient amount of dextranase (Sigma) overnight at 50°C. The resulting products were analysed using HPAEC-PAC.

The branching degree of the isolated starch was determined after isoamylase digestion according to the Luff-Schoorl method with modifications as described in [25].

The apparent amylose content was performed according to the procedure described in Hovenkamp-Hermelink et al. [26].

Starch content was determined as described by Kok-Jacon et al. [27].

Total phosphate content in the starch was determined essentially according to the method of Morrison (1964) with some modifications. About 20 mg of dry starch were suspended in 250 μl of 70% (w/w) HClO_4_ and completely charred at 250°C for 25 min. The solution was clarified by adding 50 μl 30% (w/v) H_2_O_2_ and gently boiled for 2 min. Once the solution had cooled, water was added to a final volume of 2 ml and 100 μl of the sample was transferred into a 96-well microtiter plate, followed by adding 200 μl of colour reagent [0.75% (w/v) (NH_4_)_6_M_O7_O_24_.4H_2_O, 3% (w/v) FeSO_4_.7H_2_O and 0.75% (w/v) SDS dissolved in 0.375 M H_2_SO_4_]. Absorbance at 750 nm was then measured in a Model 680 XR Microplate Reader (Bio-Rad, US), and compared to the absorption of a calibration curve to calculate the concentration in nmol PO_4_/mg starch.

Starch digestibility was determined essentially according to the method of Warren et al. [28] with some modifications. Fifty milligrams of granular starch was suspended in 4.95 mL of acetate buffer (0.2 M, pH 6, containing 200 mM CaCl_2_ and 0.5 mM MgCl_2_), followed by incubation with 50μl enzyme solution containing 10 U α-amylase (porcine pancreas, Sigma) and 5.6 U amyloglucosidase (Aspergillus niger, Megazyme) for 2h at 37°C. Aliquots (200 μL) were centrifuged and subjected to HPAEC-PAC. The amount of glucose was calculated and presented as microgram per 1 mg dry starch.

### RNA isolation and quantitative RT-PCR (qRT-PCR) analysis

Mature tubers were sampled from five plants from each clone. Samples were frozen in liquid N_2_ and stored in -80°C freezer for RNA isolation. Total RNA was extracted from potato tuber samples according to Kuipers et al. [29]. cDNA was synthesized from 1 μg of total RNA, previously treated with RNase-free DNase I (Invitrogen), by using the iScript^™^ cDNA Synthesis Kit (Bio-Rad). Quantitative real time PCR (qRT-PCR) was performed using CFX96 Real-Time PCR machine (BioRad). The total volume of each reaction was 10 μl, containing 50 ng cDNA, 3 μM of each gene-specific primer, and 5 μl SYBR Green Supermix Reagent (BioRad). All reactions were carried out in triplicate using the following thermal cycling conditions: 3 min of denaturation at 95°C, followed by 45 cycles (15 s at 95°C, 60 s at 60°C). The elongation factor 1-α (*EF1α*) gene was used as the reference to normalize gene expression across the samples because it was reported to be relatively stable in expression [30]. Target genes were expressed relative to *ef1α* using the comparative Ct method [31]. Gene-specific primers were designed with Primer-3-Plus software [32] and are listed in S1 Table.

Expression level of (engineered) *GTFB* from all transformants was determined. The relative expression level of target genes was multiplied by a factor of 10^4^ (for KDSGB and *amf*SGB series) or 10^5^ (for KDGB and *amf*GB series) and then converted to log 10. Ultimately, the resulted value (v) was used to divide transformants to different categories: undetectable (N, v = 0), low (L, 0 < v < 2), medium (M, 2≤ v < 2.5) and high (H, v ≥ 2.5) expressors.

Three or four H-expressors from each series were used for investigating expression of key genes involved in starch metabolism. These genes were glucan water-dikinase (*GWD*), starch phosphorylase (*SP*), isoamylase genes (*ISA1*, *ISA2* and *ISA3*), starch branching genes (*SBEI* and *SBEII*), starch synthase (*SSIII*), β-amylase (*BAM*) and ADP-glucose pyrophosphorylase (*AGPase*). The gene specific primers used in this study are listed in S1 Table.

### Statistical analysis

Significant differences between modified starches and control samples in the present study were assessed by one-way analysis of variance (ANOVA). The least significant difference values were calculated at 1% or 5% probability.

## Results

### (Engineered) *GTFB* is expressed in transformants

Two constructs were introduced into two potato genetic backgrounds, KD and *amf*, respectively. None of transgenic lines showed significant phenotypic differences in the above ground nor underground tissues relative to control plants.

**Fig 2:**
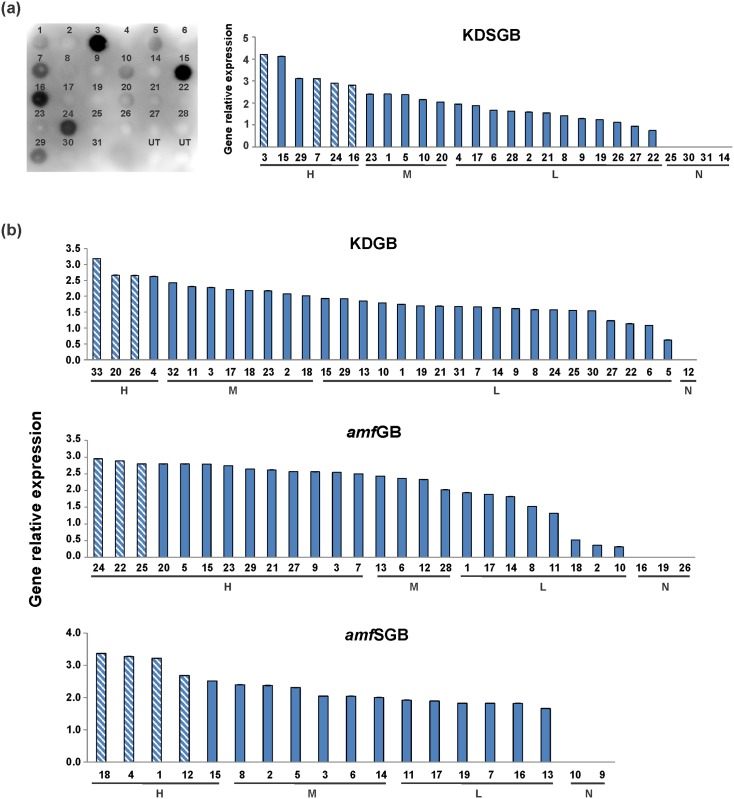
Different levels of gene expression in transgenic lines. (a) Protein accumulation level and gene expression level in KDSGB transformants. The protein accumulation and gene expression level were determined using Western dot blot with an anti-SBD antibody and qRT-PCR analysis, respectively. The number above each dot stands for the different line, while UT represents the control. The intensity of the dots shows the various protein levels. The qRT-PCR analysis was performed in triplicate for each line and the relative expression level of target genes was transformed as described in the Materials and Methods. The resulting value (v) was used to divide transformants to different categories: undetectable (N, v = 0), low (L, 0 < v < 2), medium (M, 2≤ v < 2.5) and high (H, v ≥ 2.5) expressors. (b) Distribution of the individual transformants over the classes of gene expression level in KDGB, a*mf*GB and *amf*SGB transgenic series. SGB and GB represent SBD-GTFB fusion protein and GTFB alone, respectively. KD stands for the Kardal background, while *amf* stands for the *amf* background. The striped columns indicate the lines selected for further characterization. Differences between individual transformants are due to differences in the copy number and location of the genome where the transgene was inserted.

For KDSGB series, qRT-PCR and Western dot blot analysis were performed on all transformants to examine *SBD-GTFB* expression level and the protein abundance in starch granules, respectively. The results showed that the dot intensity, which corresponds to the protein amount, was well correlated with the gene expression level (Fig 2a). For this reason, and also because no antibody is available for the detection of GTFB, transformants from other series were only investigated using qRT-PCR for gene detection and quantification (Fig 2b).

Based on the transcript level, transformants from each series were grouped into four classes respectively: undetectable (N), low (L), medium (M) and high (H) expressors. Several H-expressors from each transgenic series were selected for further detailed analysis. From the KDGB series, KDGB20, KDGB26 and KDGB33 were selected, and KDSGB3, KDSGB7, KDSGB16 and KDSGB24 were selected from the KDSGB series. In the *amf* background, *amf*GB22, *amf*GB24, *amf*GB25, *amf*SGB1, *amf*SGB4, *amf*SGB12 and *amf*SGB18 were selected for further characterization. As expected, no (engineered) *GTFB* expression was detected in the UT-KD and UT-*amf* lines (data not shown).

### Starch granule morphology is altered in transformants

**Fig 3:**
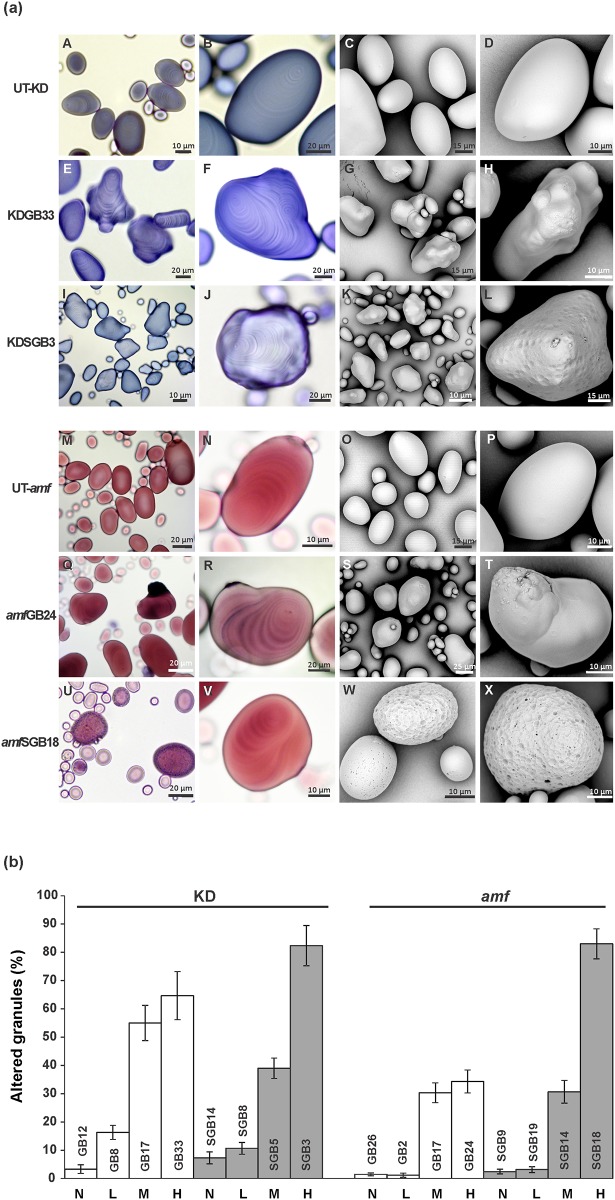
Granule morphology of starches from transformants and controls. (a) Light microscopy (LM) and scanning electron microscopy (SEM) of starch granule morphology from UT-KD (A, B, C and D), KDGB33 (E, F, G and H), KDSGB3 (I, J, K and L) UT-*amf* (M, N, O and P), *amf*GB24 (Q, R, S and T) and *amf*SGB18 (U, V, W and X). Starch granules were stained with a 20× diluted Lugol solution for light microscopy. (b) Percentage of granules with altered morphology for the various classes of gene expression level: not detected (N), low (L), medium (M) and high (H). GB and SGB are abbreviations for GTFB and SBD-GTFB, respectively. A population of 100 starch granules was counted in triplicate. The values are expressed as the mean ± SD from three independent counts.

Light microscopy (LM) and scanning electron microscopy (SEM) were used to investigate the morphology of starch granules. As shown in Fig 3, starch granule morphology was affected by the expression of the (engineered) *GTFB* in both genetic backgrounds. In the KD background, starch granules with bumpy and irregular shape were observed in starches from KDGB (Fig 3E, 3F, 3G and 3H) and KDSGB transformants (Fig 3I, 3J, 3K and 3L), while UT-KD granules showed a regular rounded form and smooth surface (Fig 3A, 3C and 3D). The growth rings in starch granules of the transformants exhibited much less concentric and regular patterns than those of the UT-KD (Fig 3B, 3F and 3J); in particular, multiple initiation sites were observed in some starch granules (Fig 3F). In some of the KDSGB starches craters were observed in the surface of the starch granules (Fig 3L).

In the *amf* background, *amf*GB starches exhibited irregular round-shaped granules (Fig 3Q and 3S), and some of these granules showed blister-like spots protruding from the surface (Fig 3T). The shape of *amf*SGB starches granule was comparable to the control (Fig 3U and 3W), however, granules were pitted with individual pores with varying size and depth (Fig 3X) rather than the smooth surface observed in the control (Fig 3P). These pores were only present on the granule surface and did not penetrate through the whole granule. Moreover, growth rings in starch granules from both *amf*GB and *amf*SGB transformants were also not distributed as evenly as in the control UT-*amf* (Fig 3R and 3V), as observed in the KD background.

To examine whether the expression level of the transgene affects the percentage of altered granules, we counted a population of 100 granules in triplicate for starches from transgenic lines that belonged to different expression classes. As shown in Fig 3b, the highest numbers of altered starch granules were observed in the H-expressors from each series. In particular, ~80% of starch granules showed morphological alteration in H-expressors (KDSGB3 and *amf*SGB18) from both KDSGB and *amf*SGB series. For the N-expressors, the frequency of altered starch granules was less than 8% in all series. Overall, a positive correlation was found between transgene expression and the percentage of altered granules regardless of the construct and background used.

### Modified starches from the *amf* background have bigger granules and higher phosphate content

**Fig 4:**
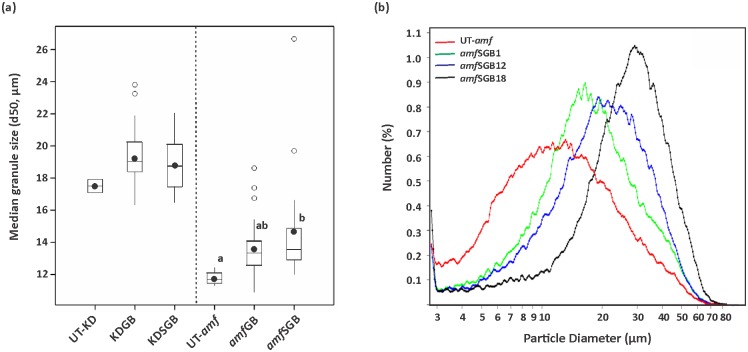
Starch granule size of starches from transgenic lines and control lines. (a) Boxplot presenting median granule size of modified starches and their respective controls. The measurements were performed on all transformants except N-expressors (lines with undetectable expression of target gene), which are 31, 23, 25 and 17 lines for KDGB, KDSGB, *amf*GB and *amf*SGB series, respectively. Boxes in the plot include values in the 25%–75% interval. Internal lines, filled circles, unfilled circles and bars represent the median, the mean, outliers and extremes, respectively. Statistical significance was analysed using one-way ANOVA. Different letters indicate statistically significant differences between means at *p* < 0.01. (b) Average particle size distribution of starches from *amf*SGB H-expressors (lines with high expression of target gene) and the control UT-*amf*. Each starch sample was analysed in duplicate.

Granule size distribution was analysed for all starches from transformants and control plants. The results showed that starches from *amf*SGB series had significantly larger granules than the control (Fig 4a, ANOVA, *p* < 0.01). As shown in Fig 4b, starch granule size increased up to two fold in H-expressors relative to the control. By contrast, no significant differences was found between starch granule size of transformants and that of the control in the KD background (Fig 4a).

**Fig 5:**
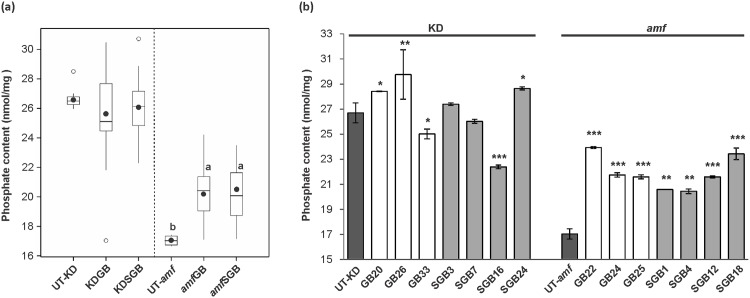
Phosphate content in starches from transgenic lines and control lines. (a) Boxplot presenting phosphate content in modified starches and their respective controls (UT-KD and UT-*amf*). The measurements were performed on starches from all transformants except N-expressors (lines with undetectable expression of target gene), which are 31, 23, 25 and 17 lines for KDGB, KDSGB, *amf*GB and *amf*SGB series, respectively. Boxes in the plot include values in the 25%–75% interval. Internal lines, filled circles, unfilled circles and bars represent the median, the mean, outliers and extremes, respectively. Statistical significance was analysed using one-way ANOVA. Different letters indicate statistically significant differences between means at *p* < 0.01. (b) Phosphate content in starches of H-expressors (lines with high expression of target gene) from each transgenic series and control lines. Columns represent mean ± SD from three independent measurements. Statistical significances between each starch sample and the control determined by using t-test (*, *p* < 0.1; **, *p* < 0.01; ***, *p* < 0.001).

In the *amf* background, modified starches showed a significant increase in phosphate content (ANOVA, *p* < 0.01), as the average amount in each series was ~19% higher than that of the control (Fig 5a). As shown in Fig 5b, the phosphate content of starches from H-expressors was significantly increased (t-test, *p* < 0.01 or 0.001) relative to that of the control line regardless of constructs. On the other hand, starches from KD background did not exhibit a significant difference in phosphate content between each transgenic series and the control (Fig 5a). No consistent changes was found in starch phosphate content between H-expressors and the control line (Fig 5b).

Amylose content and gelatinization properties were determined for all starches from transformants and their respective control. As presented in Table 1, generally, no significant differences were observed in these characteristics between modified starches and control starches (t-test). These results indicated that expression of the (engineered) *GTFB* does not affect these starch properties in both background.

**Table 1:** Overview of characteristics determined for the starches from H-expressors and controls in both backgrounds.

Clone	P (nmol/mg)	AM (%)	d50 (μm)	T_o_ (°C)	T_c_ (°C)	Δ*H* (J/g)	DE
**UT-KD**	26.7 ± 0.6	18.3 ± 0.3	17.5 ± 0.2	66.9 ± 0.2	77.4 ± 0.2	16.5 ± 0.1	3.9 ± 0.5
**KDGB20**	28.4 ± 0.0**	19.4 ± 0.2*	23.2 ± 2.5	65.9 ± 0.3	75.6 ± 0.4*	16.6 ± 0.3	4.4 ± 0.1
**KDGB26**	29.8 ± 2.0***	19.8 ± 0.0*	21.8 ± 2.1	65.9 ± 0.4	74.3 ± 0.5*	14.4 ± 0.5*	4.0 ± 0.4
**KDGB33**	25.0 ± 0.4*	19.5 ± 0.2*	19.0 ± 0.7	66.1 ± 0.3	76.0 ± 0.4	15.3 ± 0.3*	5.8 ± 0.2
**KDSGB3**	27.4 ± 0.1	19.1 ± 0.1	17.7 ± 0.7	66.1 ± 0.1	75.2 ± 0.3*	16.7 ± 0.7	4.4 ± 1.4
**KDSGB7**	26.0 ± 0.2	20.0 ± 0.3*	22.0 ± 0.6**	65.7 ± 0.4	74.4 ± 0.2**	16.0 ± 0.2	5.1 ± 0.7
**KDSGB16**	22.4 ± 0.1***	18.5 ± 0.1	18.7 ± 0.5	66.1 ± 0.1*	75.6 ± 0.5	14.6 ± 0.2**	3.2 ± 0.4
**KDSGB24**	28.6 ± 0.1**	19.2 ± 0.3	18.1 ± 0.5	66.3 ± 0.4	76.1 ± 0.2*	16.0 ± 0.2	4.9 ± 1.5
**UT-*amf***	17.0 ± 0.4	4.3 ± 0.4	11.8 ± 0.8	73.6 ± 0.1	86.3 ± 0.0	15.6 ± 0.4	6.2 ± 0.0
***amf*GB22**	23.9 ± 0.1***	3.5 ± 0.3	13.3 ± 1.0	73.1 ± 0.1	84.7 ± 0.1**	16.0 ± 0.2	5.0 ± 0.0**
***amf*GB24**	21.8 ± 0.2***	4.1 ± 0.2	14.0 ± 0.6	73.3 ± 0.3	85.2 ± 0.6	16.2 ± 0.7	5.5 ± 0.8
***amf*GB25**	21.6 ± 0.1***	4.2 ± 0.1	13.5 ± 0.7	73.7 ± 0.3	86.5 ± 0.6	18.5 ± 0.7*	4.8 ± 0.3*
***amf*SGB1**	20.6 ± 0.0**	4.3 ± 0.2	16.7 ± 0.4*	72.7 ± 0.0**	83.7 ± 0.1**	15.1 ± 0.4	7.3 ± 0.4
***amf*SGB4**	20.4 ± 0.2**	3.8 ± 0.0	13.6 ± 0.2	73.4 ± 0.1	85.3 ± 0.3	15.0 ± 0.1	5.3 ± 0.2*
***amf*SGB12**	21.6 ± 0.1***	4.1 ± 0.1	19.7 ± 0.4**	74.0 ± 0.2	86.6 ± 0.3	15.6 ± 0.3	6.2 ± 0.4
***amf*SGB18**	23.4 ± 0.5***	4.1 ± 0.0	26.7 ± 2.0*	74.5 ± 0.1*	86.4 ± 0.3	16.4 ± 0.1	6.4 ± 0.2

Data (mean ± S.D.) are the average of two or three independent measurements. P, total phosphate content; AM, apparent amylose content; d50, median granule size; T_o_ and T_c_, starch gelatinization temperature; Δ*H*, gelatinization enthalpy. DE, Dextrose Equivalent. Statistical significances between each starch sample and the control determined by using t-test (*, *p* < 0.1; **, *p* < 0.01; ***, *p* < 0.001).

Chain length distributions of starches, after complete debranching with isoamylase, were analysed using HPAEC-PAC. The results did not show significant changes in both backgrounds (data not shown). To detect the presence of linear α-1, 6 chains in starch structure, starches were treated with dextranase and the amount of glucose and isomaltose were measured. No significant differences could be detected between products obtained from modified starches and control starches in both backgrounds (data not shown).

The branching degree of starch was determined using Luff-Schoorl method and expressed in Dextrose Equivalent (DE). The DE of the modified starches from KD background ranged from 3.2 to 5.8, while the one of *amf* modified starches ranged from 5.0 to 7.3. No consistent changes in DE were observed between modified starches and controls in both backgrounds (Table 1).

### Modified starches from the KD background exhibit a higher digestibility

**Fig 6:**
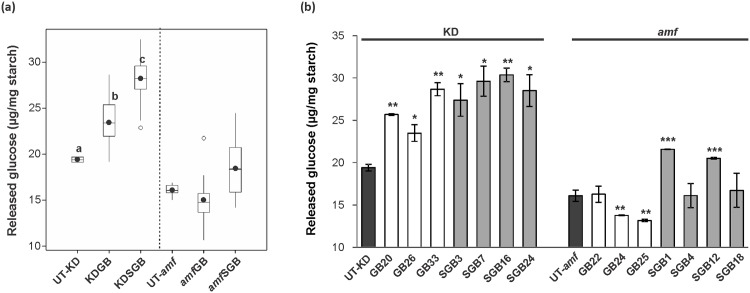
Starch digestibility analysis of starches from transformants and control lines. (a) Boxplot presenting the amount of glucose released from modified starches and their respective controls (UT-KD and UT-*amf*). Each starch sample was treated with α-amylase and amyloglucosidase and measured glucose amount in duplicate. The measurements were performed on starches from all transformants except N-expressors (lines with undetectable expression of target gene), which are 31, 23, 25 and 17 lines for KDGB, KDSGB, *amf*GB and *amf*SGB series, respectively. Boxes in the plot include values in the 25%–75% interval. Internal lines, filled circles, unfilled circles and bars represent the median, the mean, outliers and extremes, respectively. Statistical significance was analysed using one-way ANOVA. Different letters indicate statistically significant differences between means at *p* < 0.05. (b) The amount of glucose released from starches of H-expressors (lines with high expression of target gene) from each transgenic series and control lines. Columns represent mean ± SD from two independent measurements. Statistical significances between each starch sample and the control determined by using t-test (*, *p* < 0.1; **, *p* < 0.01; ***, *p* < 0.001).

To investigate whether the starch digestibility was affected by the introduction of the (engineered) GTFB, starches from all transformants and corresponding controls were treated with α-amylase and amyloglucosidase, and the amount of released glucose was measured. Overall, a significant difference in the glucose release was observed only for the starches from the KD background relative to the control UT-KD (ANOVA, *p* < 0.05), whereas no changes were found between starches from the *amf* background and the control UT-*amf*. As shown in Fig 6a, for the KDGB starches, the amount of released glucose ranged from 19 to 29 μg/mg starch, and the average value (~23 μg/mg starch) was significantly higher than that of UT-KD (~19 μg/mg starch). Moreover, ~19% and ~44% more glucose, in average, was released from KDSGB starches than from KDGB starches and the control, respectively. Starch samples of H-expressors from KD background released significantly higher amount of glucose compared to the control UT-KD (t-test, *p* < 0.05, 0.01 or 0.001), whereas there was no consistent differences in glucose release between starches from H-expressors and control in *amf* background (Fig 6b). Collectively, these results indicated that modified starches from KD background are more susceptible to degrading enzymes than the control.

### Genes involved in starch metabolism are up-regulated in transformants

**Fig 7:**
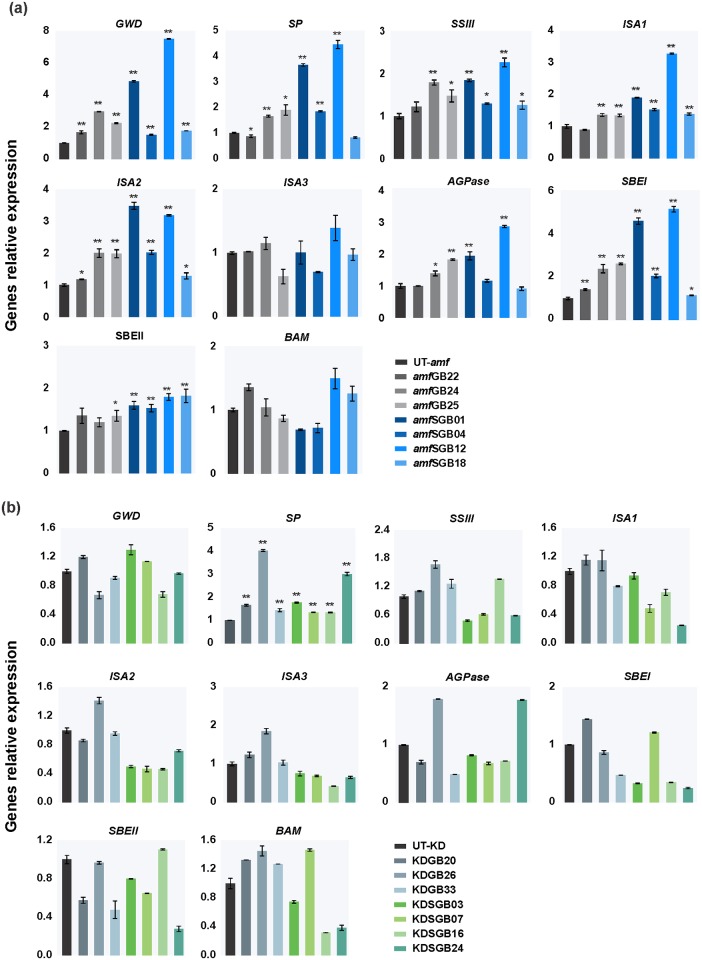
The expression level of the genes encoding key enzymes involved in starch metabolism in (a) *amf* and (b) KD potatoes. Include: glucan, water-dikinase (*GWD*), starch phosphorylase (*SP*), isoamylase (*ISA1*, *ISA2* and *ISA3*), starch branching genes (*SBEI and SBEII*), starch synthase (*SSIII*), β-amylase (*BAM*) and ADP-glucose pyrophosphorylase (*AGPase*). Three/Four H-expressors (lines with high expression of target gene) of each series and the control lines were selected and subjected to qRT-PCR analysis. Statistical significance was analysed using t-test (*p < 0.05; **p < 0.01). The values are expressed as the mean ± SD from three independent measurements.

Expression of the (engineered) *GTFB* in potato leads to alterations in starch granule morphology and an increase in granule size and starch phosphate content in *amf* transformants. To better understand these phenomena, the transcripts of key genes involved in starch metabolism were further investigate by qRT-PCR in H-expressors from each transgenic series and respective control tubers. Generally, regardless of the constructs, *amf* transformants showed a significant increase (ANOVA, *p* < 0.01 or 0.05) in the expression level of glucan, water-dikinase (*GWD*), starch phosphorylase (*SP*), isoamylase (*ISA1* and *ISA2*), starch branching enzyme I (*SBEI*) and starch synthase (*SSIII*) relative to the control, while no significant changes were observed for the other genes, such as β-amylase (*BAM*), isoamylase (*ISA3*), starch branching enzyme II (*SBEII*) and ADP-glucose pyrophosphorylase (*AGPase*) (Fig 7a). Particularly, the up-regulation of gene expression was generally higher in SGB transformants than in GB transfomants. For instance, for the *GWD* expression in *amf*SGB15 line showed an 8-fold increase compared to the control, while in *amf*GB24 the expression level was increased 3-fold For the *SP* expression, 5- and 2-fold increases were observed in these two lines respectively. On the other hand, in the KD background, no significant changes were found in the expression of these genes between transformants and the control, except for *SP* (Fig 7b). Taken together, introduction of the (engineered) GTFB into *amf* potatoes results in up-regulation of certain genes involved in starch metabolism, especially SGB. The same trends were found in KB background but the differences were smaller.

## Discussion

In this study, an (engineered) *GTFB* gene from *Lactobacillus reuteri* 121 was introduced into KD and *amf* potato plants, aiming to create liner α-1, 6-glycosidic linkages in amylopectin and amylose chains during starch biosynthesis, thus producing starches with novel structure and properties.

The results presented herein showed that differences in starch structure between modified and control starches could not be detected, suggesting that expression of the (engineered) *GTFB* in potatoes either does not introduce new chains with liner α-1, 6-glycosidic linkages into starches, or these are below detectable levels, or novel chains have been identified as an error and removed by endogenous hydrolases. The latter explanation is more likely. In other words, (engineered) GTFB could possibly introduce novel chains into amylopectin structure, while endogenous enzymes cleave off these unusual branches, leading ultimately to no changes in starch structure. Firstly, this is supported by the increase in the transcripts of starch-degrading genes (*GWD*, *ISA1*, *ISA2* and *SP*) and starch-synthesizing genes (*SSIII*, *SBEI* and *SBEII*) in transformants. Particularly, previous studies demonstrated that the removal of misplaced branches by *ISA1* and *ISA2* is essential for crystalline amylopectin synthesis [33,34], which can explain the up-regulation in transcripts of these two genes in this study. This hypothesis is in line with the model proposed by Huang et al. [21], who speculated that debranching enzymes and β-amylase remove the extra positioned branches introduced by *E*.*coli* glycogen-branching enzyme. On the other hand, while starch-degrading genes remove “error” chains, the transcripts of starch-synthesizing genes are up-regulated, thereby stimulating amylopectin synthesis for a regular granule packing.

Secondly, this hypothesis is also supported by the altered starch granule morphology from transformants, especially the uneven distribution of the growth rings, which might indicate that (engineered) GTFB introduced novel chains at first, thereby affecting the process of amylopectin assembly. Taken together, the fact that no detectable changes in starch structure via (engineered) GTFB could be observed might be due to the molecular regulation existing for starch biosynthesis. However, we cannot exclude the possibility that GTFB was not active in the potato environment. These changes in the modified starches were due to the introduction of the GTFB rather than the activity of the enzyme.

Furthermore, a higher digestibility in modified starches in KD background compared with the control has been detected. The increase may be attributed to the irregular bumpy surface of starches that provides more surface area for the binding of hydrolytic enzymes and ultimately gives rise to more hydrolysis than control starches. This result is consistent with data obtained in earlier studies, reporting that starches with a bigger granule size and smoother granule surface have lower susceptibility to enzymatic hydrolysis [35–38].

On the other hand, microscopy analyses showed varied-sized pores on the surface of starch granules from SGB transformants, which can be explained by the specific granule-binding ability of SGB fusion proteins and the deposition of them on the granule surface. The surface-attached proteins might be released later on, consequently generating the porous surface. This is further supported by the observation that the percentage of altered granules increases in transgenic plants with higher SGB expression (Fig 3b). This result is consistent with previous studies showing that surface pores were visible after removing the protein from granule surface in cereals [39,40]. Moreover, the presence of amylose might explain the shallower pores in KDSGB starches compared to that in *amf*SGB. Similar observations have been made by introducing other granule-bound proteins into potatoes [21,41,42].

In the *amf* background, an increase in phosphate content of modified starches is likely to be a result of the up-regulation in *GWD* expression, which has proven to be mainly responsible for the starch phosphate content [43,44]. In addition, one could expect to observe the differences in starch composition or properties because of the changes in granule morphology, granule size and phosphate content in modified starches. However, no significant changes in amylose/amylopectin ratio, gelatinization temperatures or starch digestibility were detected between modified starches and the control starch. This phenomenon can be a consequence of multiple combined effects caused by all these alterations, each of which is difficult to quantify.

In summary, this study gives further insights into the complex functions and interplay between starch metabolic enzymes in storage starches, highlighting the molecular regulation of amylopectin assembly, and providing a further step toward understanding the biosynthesis of storage starches. Further investigation is required to decipher the precise mechanism of starch granule formation and to identify determinants that are responsible for this process. Ultimately, our results re-inforce the notion that genetic modification is not only a powerful tool for uncovering and exploiting starch biogenesis in plants, but also an effective and economical manner for tailoring starches with improved properties for industry applications like in this case the enhanced release of glucose from KD starches.

## Supporting Information

S1 Fig Schematic representation of pBIN19/SBD2-*XbaI* vector. This construct was modified based on pBIN19/SBD2 [24] by adding an *XbaI* restriction site. RB and LB represent right and left borders, respectively. SBD, LK, Kan and 3’NOS stand for starch binding domain of cyclodextrin glycosyltransferase from *B*. *circulans*, linker, kanamycin resistant gene and NOS terminator, respectively. *Hpa*I, *Sal*I and *Xba*I are restriction enzymes.
(PDF)

S1 Table The qRT-PCR primer sequences of genes of interest and reference gene. (PDF)
