# Supplementary material for: Expression of an (Engineered) 4,6-α-Glucanotransferase in Potato Results in Changes in Starch Characteristics
Source: PLoS One. 2016 Dec 2;11(12):e0166981. doi: 10.1371/journal.pone.0166981 (PMC5135068; doi:10.1371/journal.pone.0166981)
Supplement: S1 Fig — This construct was modified based on pBIN19/SBD2 [24] by adding an XbaI restriction site. RB and LB represent right and left borders, respectively. SBD, LK, Kan and 3’NOS stand for starch binding domain of cyclodextrin glycosyltransferase from B. circulans, linker, kanamycin resistant gene and NOS terminator, respectively. HpaI, SalI and XbaI are restriction enzymes. (PDF) [file pone.0166981.s001.pdf]

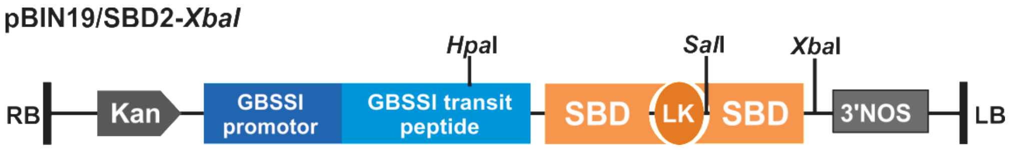

S1 Fig. Schematic representation of pBIN19/SBD2-*XbaI* vector. This construct was modified based on pBIN19/SBD2 (Ji et al. 2004) by adding an *XbaI* restriction site. RB and LB represent right and left borders, respectively. SBD, LK, Kan and 3'NOS stand for starch binding domain of cyclodextrin glycosyltransferase from *B. circulans*, linker, kanamycin resistant gene and NOS terminator, respectively. *Hpa*I, *Sal*I and *Xba*I are restriction enzymes.
