# Supplementary material for: Expression of an (Engineered) 4,6-α-Glucanotransferase in Potato Results in Changes in Starch Characteristics
Source: PLoS One. 2016 Dec 2;11(12):e0166981. doi: 10.1371/journal.pone.0166981 (PMC5135068; doi:10.1371/journal.pone.0166981)
Supplement: S1 Table — (PDF) [file pone.0166981.s002.pdf]

S1 Table. The qRT-PCR primer sequences of genes of interest and reference gene.

Gene	Gene ID	Forward primer 5'-3'	Reverse primer 5'-3'	Reference
GWD	AY027522.1	gggtggtcttttgcgtgatt	tgtagcccatgcagtttgca	[1]
BAM	XM_006340834.2	cctccttccccactgatctg	gaaacgtcggtgattcctgc	[1]
AGPase	X55155	gctgggacccgactttatcc	cgggaatgtcaatcagacgat	[2]
SP	X52385	caggaaccagatgctgctctt	catagccccatgctgggtagt	[3]
ISA1	102577466	ggcaaatggagaggacaaca	atgggaacaccttgggaaac	[4]
ISA2	102577931	ttatccttccgccacctc	cttcaactggagttcccttct	[4]
ISA3	102577824	gacgcttgcccttcattc	ctcctgtgcggttcttctgt	[5]
SBEI	102596498	ccgagccccacgaatctat	ggctcagagctgctcatgc	[6]
SBEII	102590711	actcgcaactgatgtggatg	ggcattccgctaacatcttc	[7]
SSSIII	102577674	cacaggaggtgtctggaaacc	tggaacttgtgaaggtgaggc	[8]
EF1α	AB061263	attggaaacggatatgctcca	tccttacctgaacgcctgtca	[9]

References:

1. Chen X, Song B, Liu J, Yang J, He T, et al. (2012) Modulation of gene expression in cold-induced sweetening resistant potato species Solanum berthaultii exposed to low temperature. Molecular genetics and genomics 287: 411-421.
2. Muller-Rober BT, Kossmann J, Hannah LC, Willmitzer L, Sonnewald U (1990) One of two different ADP-glucose pyrophosphorylase genes from potato responds strongly to elevated levels of sucrose. Mol Gen Genet 224: 136-146.
3. Zhang H, Liu X, Liu J, Ou Y, Lin Y, et al. (2013) A novel RING finger gene, SbRFP1, increases resistance to cold-induced sweetening of potato tubers. FEBS letters 587: 749-755.
4. Bustos R, Fahy B, Hylton CM, Seale R, Nebane NM, et al. (2004) Starch granule initiation is controlled by a heteromultimeric isoamylase in potato tubers. Proceedings of the National Academy of Sciences of the United States of America 101: 2215-2220.
5. Ferreira SJ (2011) Transcriptome based analysis of starch metabolism in Solanum tuberosum.
6. Safford R, Jobling SA, Sidebottom CM, Westcott RJ, Cooke D, et al. (1998) Consequences of antisense RNA inhibition of starch branching enzyme activity on properties of potato starch. Carbohydrate Polymers 35: 155-168.
7. Jobling SA, Schwall GP, Westcott RJ, Sidebottom CM, Debet M, et al. (1999) A minor form of starch branching enzyme in potato (Solanum tuberosum L.) tubers has a major effect on starch structure: cloning and characterisation of multiple forms of SBE A. Plant Journal 18: 163-171.
8. Abel GJ, Springer F, Willmitzer L, Kossmann J (1996) Cloning and functional analysis of a cDNA encoding a novel 139 kDa starch synthase from potato (Solanum tuberosum L.). Plant J 10: 981-991.
9. Nicot N, Hausman J-F, Hoffmann L, Evers D (2005) Housekeeping gene selection for real-time RT-PCR normalization in potato during biotic and abiotic stress. Journal of experimental botany 56: 2907-2914.
